# Supplementary material for: Large-deviations of disease spreading dynamics with vaccination
Source: PLoS One. 2023 Jul 10;18(7):e0287932. doi: 10.1371/journal.pone.0287932 (PMC10332629; doi:10.1371/journal.pone.0287932)
Supplement: S1 Appendix — (PDF) [file pone.0287932.s001.pdf]

Supporting Information to: Large-deviations of disease spreading dynamics with vaccination

Yannick Feld[1*], Alexander K. Hartmann[1]

1 Institut für Physik, Carl von Ossietzky Universität Oldenburg, 26111 Oldenburg, Germany

* yannick.feld@uni-oldenburg.de

Additional pdfs and rate functions.
In Fig 1 we show the pdfs for the adaptive high-degree heuristics measured at their respective critical vaccination doses n_v.

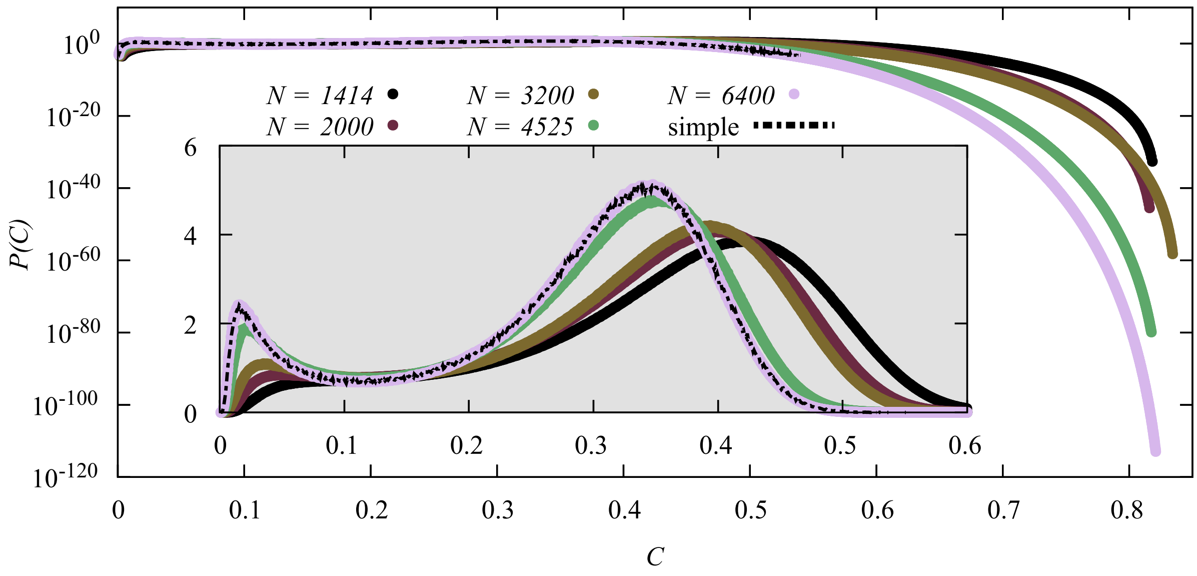

Fig 1. Probability density of the cumulative fraction C of infections for different system sizes N at their critical vaccination doses n_v^c with the adaptive high-degree vaccination strategy as measured by the large-deviation algorithm. It also includes simple sampling data for the largest system size. Linear scale in the inset.

The general shape of the resulting pdfs is very similar to the ones from the random heuristics, however the position of the second peak is shifted more towards the right and the height of the first peak is much lower. This fits well with the observation that the average $\overline{C}$ at the critical vaccination dose n_v^c is higher for the high degree heuristics (see Fig 1 in the paper) than for the random heuristics.

Looking at the rate functions shown in Fig 2 we also see a similar picture.

Again it seems like the shape to the left of the second minimum, i.e., for about $C < 0.3$, converges to a limiting shape for $N \to \infty$. Beyond the minimum though no clear convergence is visible until we shift the functions in such a way that their minimum occurs at the same position (not shown).

We also fitted an exponential function to the position of the minimum, but the fit does not work as well as before. Note that for each system size we only look at a single, arbitrarily drawn network, so some discrepancies are to be expected and it is rather remarkable that those were not noticeable for the random vaccination heuristics, even though we used the exact same networks for each vaccination strategy.

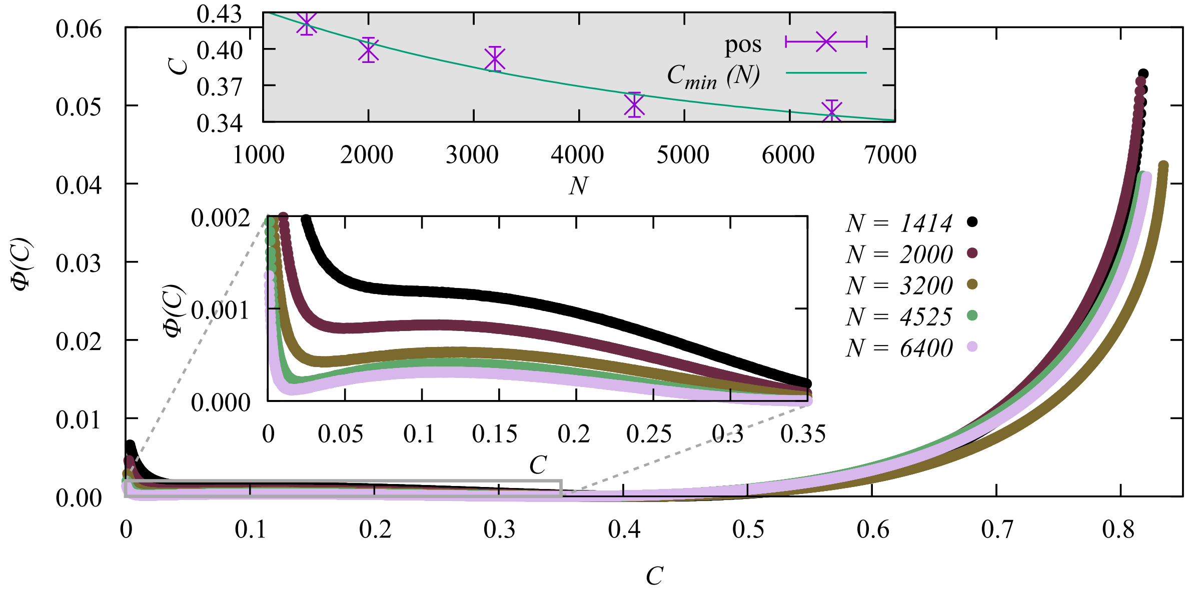

Fig 2. Rate functions $\Phi(C)$ for the adaptive high-degree vaccination heuristics for different system sizes N at their respective critical vaccination. The gray inset shows the position of the minimum of the rate function as function of N as well as the fit according to Eq (5) in the paper with $a = 0.15(3)$, $b = 0.00026(26)$ and $C_{\min}^{\infty} = 0.32(6)$, which means the fit has rather large errors. The other inset shows a zoom for better visibility.

For the sake of completion we show the measured probability densities for the non-adaptive high-degree heuristics in Fig 3. The general shapes of the pdfs for the two high-degree heuristics are very similar.

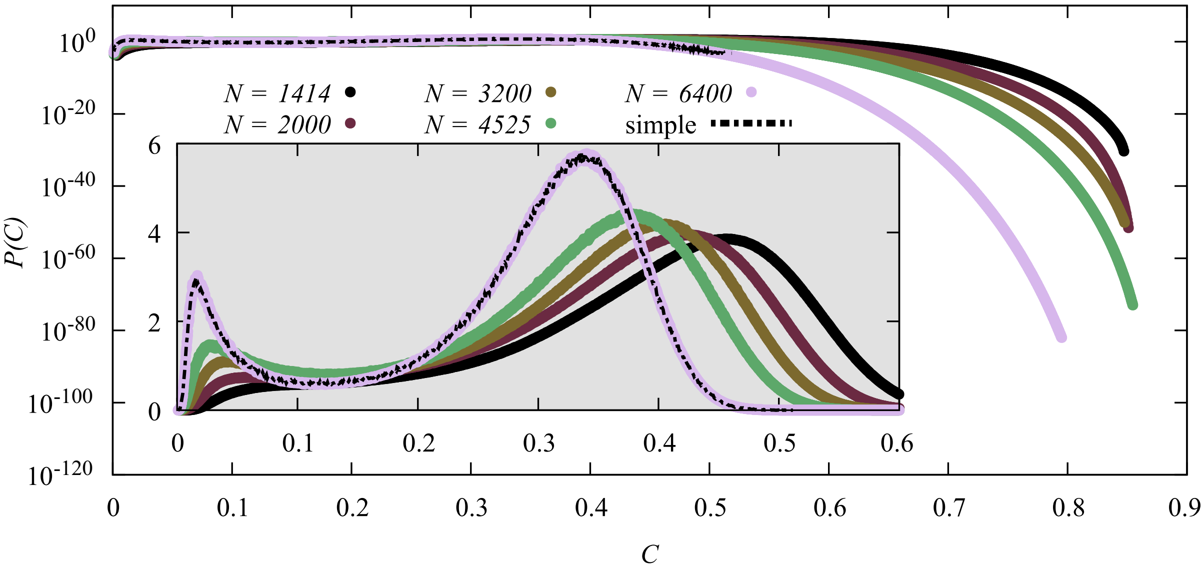

Fig 3. Probability density of the cumulative fraction C of infections for different system sizes N at their critical vaccination doses n_v^c with the non-adaptive high-degree vaccination strategy as measured by the large-deviation algorithm. It also includes simple sampling data for the largest system size. Linear scale in the inset.

The respective rate functions are shown in Fig 4 and exhibit a similar behavior. This time, however,

shifting the functions in such a way that their positions of the minima agree (not shown) does not lead to a complete monotonous pattern, as the function for $N = 6400$ now is located in between those for $N = 3200$ and $N = 4525$.

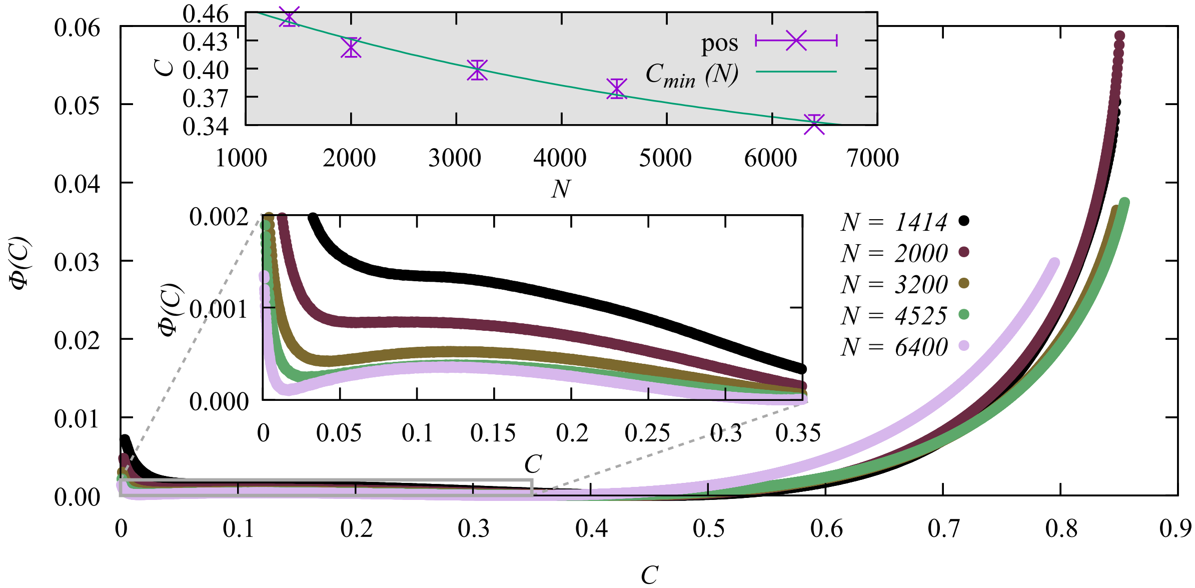

Fig 4. Rate functions $\Phi(C)$ for the non-adaptive high-degree vaccination heuristics for different system sizes N at their respective critical vaccination. The gray inset shows the position of the minimum of the rate function as function of N as well as the fit according to Eq (5) in the paper with $a = 23(7)$, $b = 0.00019(15)$ and $C^{\infty}_{\text{min}} = 0.28(9)$, which means the fit has rather large errors. The other inset shows a zoom for better visibility.
