# Supplementary figures and images for: Large-deviations of disease spreading dynamics with vaccination
Source: PLoS One. 2023 Jul 10;18(7):e0287932. doi: 10.1371/journal.pone.0287932 (PMC10332629; doi:10.1371/journal.pone.0287932)

adaptive high degree

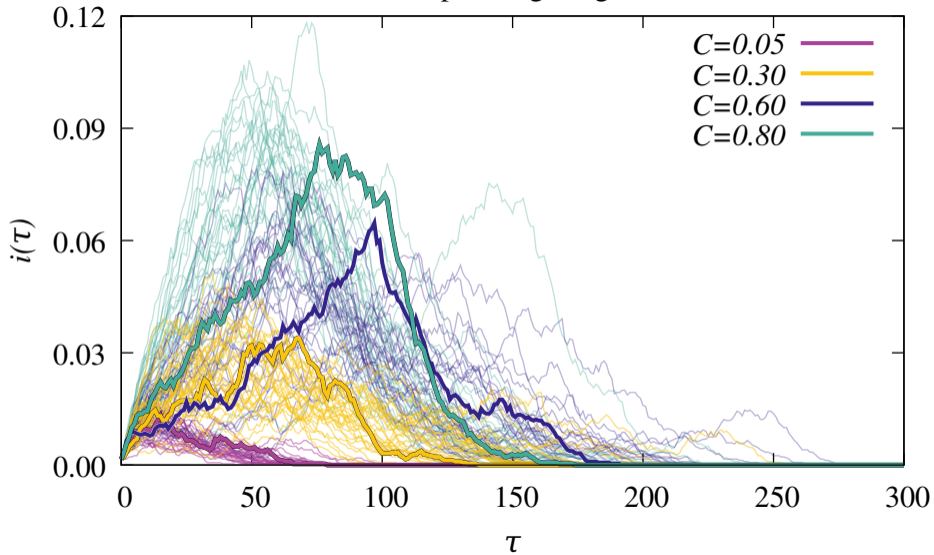

Supplement: S1 Data — (GZ) [file pone.0287932.s002.gz › DATA/fig11/adaptive/adaptive_curves.pdf]

non-adaptive high degree

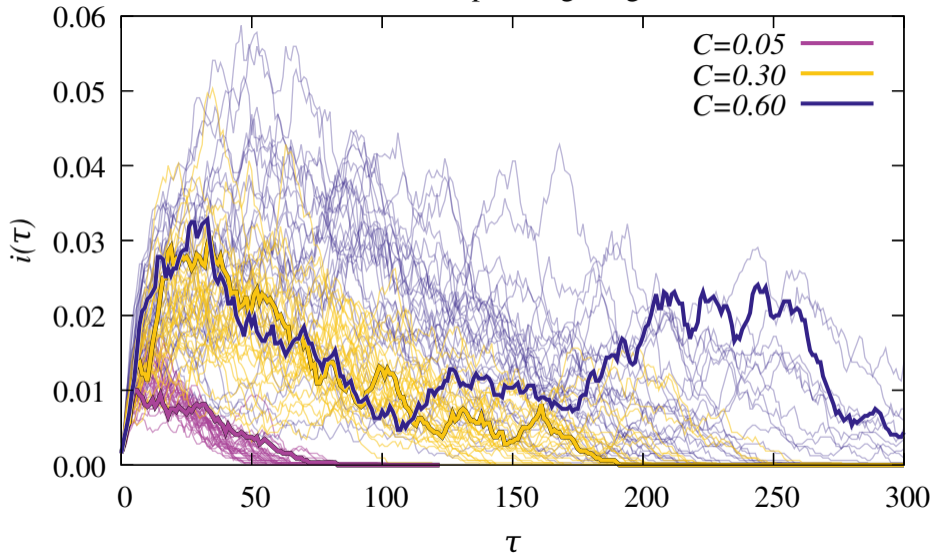

Supplement: S1 Data — (GZ) [file pone.0287932.s002.gz › DATA/fig11/non-adaptive/non_adaptive_curves.pdf]

non-adaptive high degree

$V_i(C_1, C_2)$

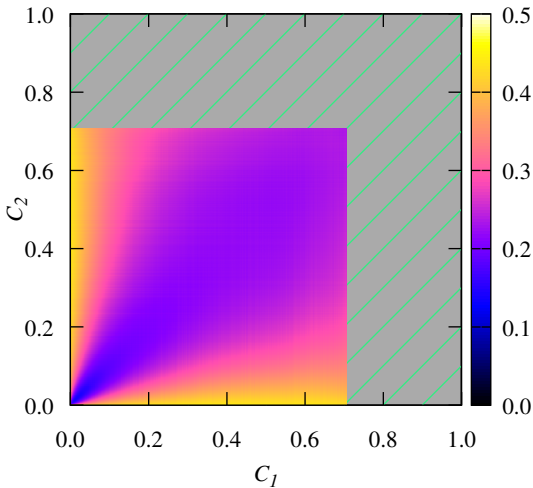

Supplement: S1 Data — (GZ) [file pone.0287932.s002.gz › DATA/fig10/non_adaptive/disparity_hd2.pdf]

random

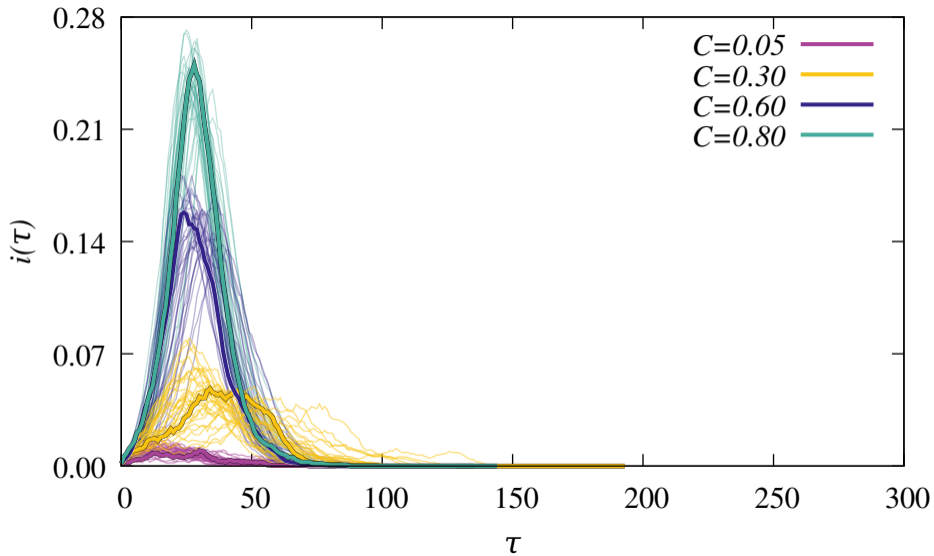

Supplement: S1 Data — (GZ) [file pone.0287932.s002.gz › DATA/fig11/random/random_curves.pdf]

non-adaptive high degree

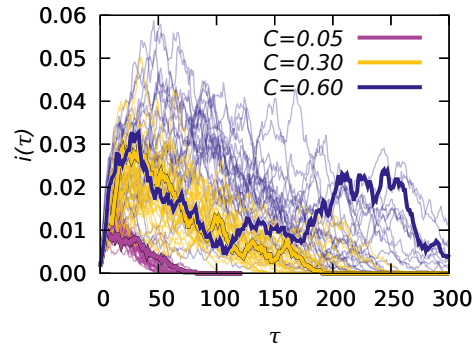

adaptive high degree

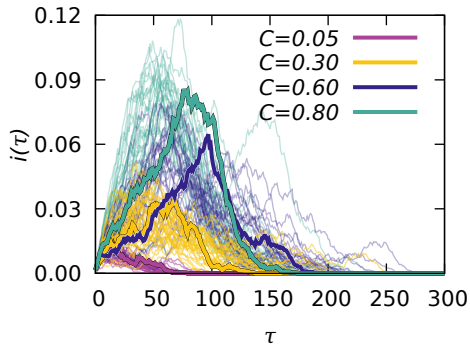

random

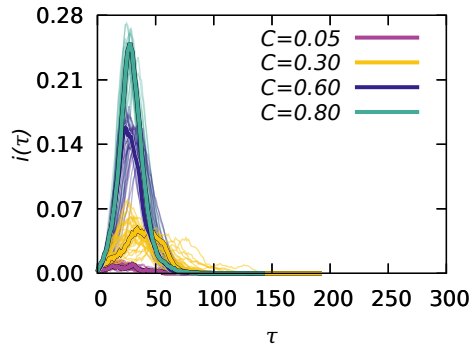

Supplement: S1 Data — (GZ) [file pone.0287932.s002.gz › DATA/fig11/fig11_curves.pdf]

random

$V_i(C_1, C_2)$

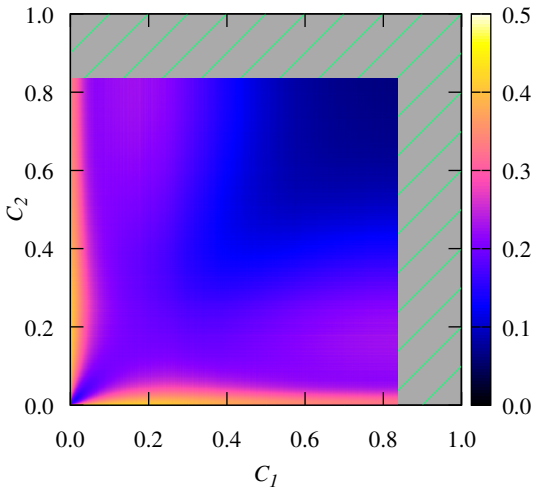

Supplement: S1 Data — (GZ) [file pone.0287932.s002.gz › DATA/fig10/random/disparity_rand.pdf]

adaptive high degree

$V_i(C_1, C_2)$

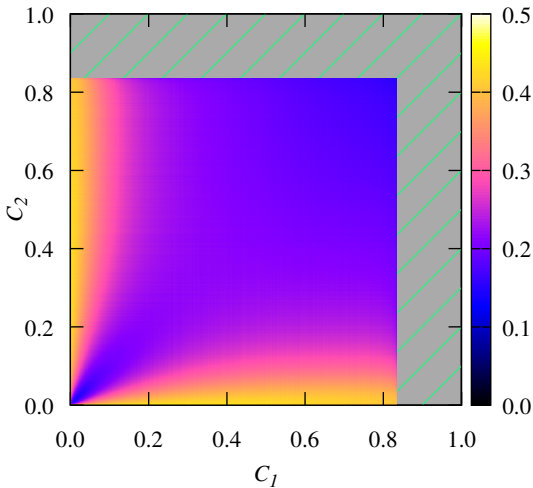

Supplement: S1 Data — (GZ) [file pone.0287932.s002.gz › DATA/fig10/adaptive/disparity_hd.pdf]
