# Supplementary material for: Large-deviations of disease spreading dynamics with vaccination
Source: PLoS One. 2023 Jul 10;18(7):e0287932. doi: 10.1371/journal.pone.0287932 (PMC10332629; doi:10.1371/journal.pone.0287932)
Supplement: Attachment — Submitted filename: revison_letter_to_referee.pdf [file pone.0287932.s007.pdf]

Answer Letter

Dear Referee,

Thank you for your quick and valuable feedback

We have included the suggested references and have briefly discussed the analogy to the chemical reaction networks in the introduction. The corresponding paragraph reads:

"Note that the SIR process can be understood as special case of chemical reaction networks, where the fractions of susceptible, infected and recovered correspond to the concentrations of chemical species [28, 29]. In these networks the nodes typically denote the species while the edges describe the chemical reactions. Although diffusion of species is often included in the networks, this and all other reactions are usually described on mean-field level, similar to what was done for the first SIR models. "

We also added a note in the summary part where we point to the results that exist for chemical reaction networks. It reads: "Note that some results exist for chemical reaction networks [28]. Since they are treated typically on mean-field level, the corresponding distribution functions usually have a simple structure, like a multidimensional simple Gaussian. Still, there are also known cases where distributions with multiple peaks have been found, like for a simple four-species reaction network [29]. Therefore the multi-modal structure of the distributions we observe might not be due to the complex underlying network, although all details of $P(C)$ will certainly depend on the network structure."

We didn't do a more detailed comparison as that would have been quite complicated. We are not aware an analytical calculation of the distribution for a chemical network where the transitions match with the SIR model, i.e, we have 3 chemical species S_1, S_2, S_3 where we have the transitions $S_1 + S_2 \to 2S_2$ with some rate λ and from $S_2 \to S_3$ with some rate β, such that the comparison makes sense. The systems we know from literature are to dissimilar to the SIR model, therefore we opted for the more general comparison stated above.

Kind regards,
Y. Feld and A.K. Hartmann
